# Utility of Diffusion Weighted Magnetic Resonance Imaging in Early Detection and Staging of Acute Pancreatitis: Correlation with Revised Atlanta Classification

**DOI:** 10.2174/0115734056425809251202131433

**Published:** 2026-01-30

**Authors:** Reem M Elkady, Shaimaa H. Bakr, Hassan I. Megally, Inas Abdullah Barakat, Walaa Alsharif, Fahad H. Alhazmi, Maisa Elzaki, Sultan Abdulwadoud Alshoabi, Amirah Alsaedi, Awadia Gareeballah, Mohamed ELmaghraby, Wael A. Abbass

**Affiliations:** 1 Department of Diagnostic Radiology, Faculty of Medicine, Assiut University, Assiut, Egypt; 2 Department of Diagnostic Radiology, College of Applied Medical Science, Taibah University, Madinah, Saudi Arabia; 3 Department of Gastroenterology, Faculty of Medicine, Mansoura University, Mansoura, Egypt; 4 Department of Internal Medicine, College of Medicine, Taibah University, Madinah, Saudi Arabia; 5 Faculty of Medicine, Mansoura University, Mansoura, Egypt; 6 Department of Internal Medicine, Faculty of Medicine, Assiut University, Assiut, Egypt

**Keywords:** MRI, Acute pancreatitis, Revised atlanta classification, DWI, Necrotizing pancreatitis, ADC values

## Abstract

**Background:**

Acute pancreatitis (AP) is associated with a high mortality rate that is directly related to its severity. Limited research has been conducted on the role of DWI-MRI in the diagnosis and staging of acute pancreatitis as it pertains to the revised Atlanta classification. The objective of this study was to examine the role of diffusion-weighted (DW) magnetic resonance imaging (MRI) in early diagnosis and staging of acute pancreatitis in correlation to the revised Atlanta classification.

**Methods:**

According to the revised Atlanta classification, a prospective assessment was performed to examine the correlation between DW MRI and apparent diffusion coefficient (ADC) values with the severity of acute pancreatitis (AP) in a sample of 34 patients diagnosed with AP.

**Results:**

The mean ADC value of mild edematous pancreatitis was 1.14±0.06x10-3 mm^2^/sec, moderate edematous pancreatitis was 1.18±0.16x10-3 mm^2^/sec, severe necrotizing pancreatitis was 1.99±0.06x10-3 mm^2^/sec, and that of the normal pancreas was 1.54±0.05 x10-3 mm^2^/sec. Based on the revised Atlanta classification, there was a significant difference between the ADC values of normal pancreas and acute, severe, and mild/moderate pancreatitis, while there was no significant difference between mild and moderate pancreatitis cases. ROC analysis yielded high accuracy in differentiating normal pancreas from acute pancreatitis and severe pancreatitis from non-severe pancreatitis (AUC=0.827 and 0.870, respectively).

**Discussion:**

In the current study, the qualitative assessment of DWI images indicated that all cases of mild acute pancreatitis (AP) displayed true diffusion restriction, while facilitated diffusion was observed in 80% of patients diagnosed with necrotizing pancreatitis. Our findings have validated the outcomes of earlier research regarding the average ADC values of both the healthy and acutely inflamed pancreas. According to the Revised Atlanta Classification, DWI has the ability to assist in the prompt diagnosis of acute pancreatitis and to differentiate mild forms from severe ones.

**Conclusion:**

DW-MRI using both qualitative and quantitative methods provides a concise, safe, and radiation-free imaging method for early detection and assessing the severity of acute pancreatitis.

## 1 INTRODUCTION

Acute pancreatitis (AP) constitutes a non-infectious inflammation of the pancreas, exhibiting a wide variety of clinical presentations and levels of severity. The morbidity and mortality rates associated with AP fluctuate between 10% and 40% among various populations, depending on the severity of the condition and the comorbidities present in patients [1-4]. Diagnosis is determined by a combination of upper abdominal pain, increased serum pancreatic enzyme levels, and radiological findings indicative of pancreatic inflammation [5]. Prompt identification and accurate evaluation of disease severity are essential for efficient clinical management. Moreover, the complications associated with acute pancreatitis are closely linked to patient prognosis [3]. In 2012, the AP Classification Working Group revised the Atlanta Classification, which currently classifies AP into two categories: interstitial edematous pancreatitis (IEP) and necrotizing pancreatitis (NP). Additionally, it established two phases of the disease (early and late). The severity of the disease is categorized as mild, moderately severe, or severe [6]. Computed tomography (CT) is generally the preferred method for diagnosing acute pancreatitis (AP) along with its local complications [7]. Despite the high sensitivity and specificity of CT, its diagnostic efficacy is limited in the context of early and mild interstitial cases [8, 9]. Furthermore, CT is not suitable for patients with contraindications to iodinated contrast media, as it has been shown to exacerbate the condition and deteriorate the renal failure frequently associated with acute pancreatitis [10, 11]. Magnetic resonance imaging (MRI) provides benefits compared to CT in detecting mild disease, peripancreatic fat infiltration, and evaluating abnormalities in the pancreatic and biliary ducts [12]. Diffusion-weighted MRI (DW-MRI) is a valuable sequence in emergency settings due to its brief acquisition time, lack of radiation exposure, and high image resolution [13]. In diffusion-weighted magnetic resonance imaging (DW-MRI), the apparent diffusion coefficient (ADC) values serve as quantitative parameters that indicate the microenvironment of water molecule diffusion. Studies have demonstrated that the restriction of DW-MRI signals and the decrease in ADC values can identify inflammatory alterations in pancreatic tissue, even in individuals who exhibit no visible abnormalities on CT scans [14]. Nevertheless, there have been limited studies examining the significance of DWI-MRI in diagnosing and staging acute pancreatitis (AP) in relation to clinical classifications. The objective of this study is to investigate the utility of DW-MRI for the early diagnosis and staging of AP, correlating imaging findings with the severity of the disease based on the revised Atlanta classification.

## 2 MATERIALS AND METHODS

### 2.1 Study Design

Thisprospective studywasconductedattheradiology department, Assiut University Hospital, over a period of one year.

### 2.2 Ethical Considerations

Approval from the institutional ethical committee was obtained (reference number: 04-2025-201234), and all procedures were carried out in accordance with the Declaration of Helsinki 1975, updated in 2013 (http://ethics.iit.edu/ecodes/ node/3931), and all relevant laws and standards. Informed consent was secured from each participant.

### 2.3 Study Population

#### 2.3.1 Sample Size

According to the available references, the global incidence rate for acute pancreatitis is approximately 34 cases per 100,000 persons/year. We calculated the sample size based on this percentage.

#### 2.3.2 Inclusion Criteria

A total of 34 patients diagnosed with AP were included, each presenting with at least two of the three defining characteristics: characteristic abdominal pain, elevated serum amylase or lipase levels at least three times above the normal upper limit, and specific Imaging features of AP on ultrasound or contrast-enhanced CT (CECT) scan.

#### 2.3.3 Exclusion Criteria

The exclusion criteria encompassed a history of chronic pancreatitis, prior pancreatic surgery, patients who were severely ill or uncooperative, and general contraindications for MRI examinations. A control group was formed with 20 patients matched for age and gender, all of whom displayed normal findings on abdominal MRI.

### 2.4 Data Collection

According to the revised Atlanta classification (Table **1**), patients were classified as experiencing mild, moderately severe, or severe acute pancreatitis, depending on whether local and systemic complications and organ failure were present or absent.

### 2.5 Data Acquisition

The MRI examination was conducted utilizing a 1.5 Tesla MR scanner (Achieva; Philips Medical Systems) equipped with an abdominal phased array Torso coil. All patients underwent an MRI examination one week after the onset of the attack. The protocols that follow were adopted: coronal turbo spin-echo T2-weighted imaging, axial turbo spin-echo T2-weighted imaging, axial T1 FFE-weighted imaging, and axial T2-SPAIR weighted sequence. DW-MRI was obtained in the axial plane before the administration of the contrast medium utilizing a single-shot spin-echo echo-planar imaging sequence with b values of (0, 200, 400, and 800 s/mm^2^). All sequence parameters are detailed in Table **2**. ADC maps were produced by Philips Medical Systems, and the ADC value was assessed for each segment of the pancreas.

Complementary dynamic contrast-enhanced MRI was performed for all cases; axial pre- and post-contrast fat-saturated T1 THRIVE images were obtained immediately after manually injected gadolinium chelate at a dose of 0.1 mmol/kg of body weight (maximum, 10 mL), and images were obtained sequentially at 30, 70, 120 sec and at 4 minutes.

### 2.6 Image Analysis

A radiology consultant with ten years of expertise in reporting abdominal MRI examined all images for DWI and ADC analysis. The radiologist was kept blind to the clinical and laboratory results. The analysis of MR images was conducted as follows [15]:

- The pancreas was assessed for enlargement, defined as an anterior–posterior diameter of ≥ 3 cm on axial images.
- The signal intensity of the pancreas was evaluated across various sequences.
- The degree of contrast medium uptake by the pancreatic parenchyma was categorized as <30%, 30–50%, or >50%.
- The presence of peripancreatic fluid collections was noted.
- Additional extra-pancreatic findings were documented, including biliary stones, extrahepatic biliary dilation, portal venous thrombosis, splenic vein thrombosis, or arterial (pseudo) aneurysms.

DWI images were qualitatively interpreted by evaluating the signal intensity of the pancreas on the diffusion image as follows:

- Low signal intensity with high signal in the corresponding ADC maps (facilitated)
- High signal intensity with a lowering of the signal in the corresponding ADC maps (restricted)

The ADC maps were interpreted quantitatively as follows: The pancreas was segmented into the head, body, and tail. For each patient, ADC values from these segments were captured by employing the largest feasible region of interest (ROI), approximately 35.5 cm^2^. The ADC values were measured three times for each segment, with different ROIs applied in various locations within the segment. The average of these measurements was then determined. In necrotizing cases, the ROI was positioned in the necrotic area, which was considered the ADC value for such cases. Areas with peripancreatic fluid, the pancreatic duct, and artifacts were excluded from the ROI.

### 2.7 Statistical Analysis

All statistical analyses were conducted utilizing Microsoft Excel 2010 and SPSS (Statistical Package for Social Science) version 15 for Microsoft Windows. Data were statistically characterized in terms of range, mean, standard deviation (SD), median, and frequencies. The quantitative variables across the examined groups were analyzed using the Student t-test. The exact test was utilized when the anticipated frequency fell below 5. Statistical significance was established at *P* ≤ 0.05. Accuracy was represented by sensitivity, specificity, positive predictive value, negative predictive value, and overall accuracy. Receiver operating characteristic (ROC) analysis was employed to determine the optimal cut-off value for the studied groups.

## 3 RESULTS

Among all patients participating in the study, 29.4% were male and 70.6% were female. Patients had a mean age of 41.4±16.9 years (with a range of 7–74 years), and there was no statistically significant difference in age or sex between the acute pancreatitis (AP) group and the control groups. The causes of AP were identified as gallstones in 58.8% of the cases, post-endoscopic retrograde cholangiopancreatography (ERCP) in 8.8%, alcohol consumption in 2.9%, trauma in 2.9%, hypertriglyceridemia in 2.9%, and idiopathic in 23.5% of the cases. According to the updated Atlanta classification, patients were classified as having mild (29.4%), moderate (58.8%), or severe AP (11.8%) as illustrated in Fig. (**1**).

In conventional MRI, most cases (64.7%) displayed no signal alteration on T1-weighted imaging (T1WI). On T2-weighted imaging (T2WI), 70.6% of cases demonstrated a hyperintense signal, whereas on T2 Short Tau Inversion Recovery (SPAIR), 94.1% of patients exhibited a hyperintense signal in comparison to the liver. Interstitial edematous acute pancreatitis (AP) was identified in 85.3% of patients, presenting with delayed homogeneous enhancement. Conversely, the remaining 4.7% were diagnosed with necrotizing acute pancreatitis, revealing areas that were devoid of enhancement in every phase. As regards the visual analysis of DWI, 88.2% of the cases displayed high signal intensity on DWI images alongside low signal on ADC maps, which indicates restricted diffusion. Of these cases, 97% fulfilled the criteria for Interstitial edematous pancreatitis (IEP), As shown in Fig. (**2A**-**D**), whereas 3% satisfied the criteria for necrotizing pancreatitis (NP). In contrast, 11.8% of the cases showed low signal on DWI images and high signal on the ADC map (indicating facilitated diffusion), with all these cases meeting the criteria for NP as shown in Fig. (**3A**-**D**).

The quantitative analysis of DWI indicated the following average ADC values: for pancreatic heads, it was 1.14±0.09 × 10^-3 mm^2/sec; for pancreatic bodies, it was 1.18±0.21 × 10^-3 mm^2/sec; and for pancreatic tails, it was 1.2±0.15 × 10^-3 mm^2/sec. Additionally, the mean ADC values based on disease severity were as follows: the mean ADC value for mild edematous pancreatitis was 1.14±0.06 × 10^-3 mm^2/sec, for moderate edematous pancreatitis it was 1.18±0.16 × 10^-3 mm^2/sec, for severe necrotizing pancreatitis it was 1.99±0.06 x10-3mm^2^/sec, and for a normal pancreas, it was 1.54±0.05 × 10^-3 mm^2/sec, as shown in Fig. (**4**). The comparison of ADC values across various groups, categorized according to the updated Atlanta Classifications, demonstrated a statistically significant difference between normal pancreas and acute pancreatitis cases (*p* < 0.001), as well as between severe pancreatitis and mild/moderate pancreatitis (*p* < 0.001). Nevertheless, no significant difference was found between mild and moderate pancreatitis cases (*p* = 0.349), (Table **3**).

The receiver operating characteristic (ROC) analysis indicated a significant diagnostic accuracy for differentiating a normal pancreas from acute pancreatitis (AP) with an AUC of 0.827, and for distinguishing severe pancreatitis from non-severe cases (AUC = 0.870). Conversely, it exhibited low accuracy in differentiating mild from moderate cases of AP (AUC = 0.635) as shown in Fig. (**5A**-**C**). The AUC values, cutoff thresholds, sensitivity, specificity, positive predictive value (PPV), negative predictive value (NPV), and diagnostic accuracy are detailed in Table **4**.

## 4 DISCUSSION

The clinical presentation of acute pancreatitis (AP) can range from mild to severe, and in some cases, it can be life-threatening. Prompt diagnosis and precise assessment of the severity of the disease are crucial for effective clinical management [5]. The capability for early diagnosis and the practicality of MRI for patients with impaired renal function have integrated MRI into the clinical management framework of AP in recent years. Additionally, recent advancements in MRI technology have enabled ultrafast imaging while minimizing imaging artifacts caused by motion [16-18]. In the current study, the qualitative assessment of DWI images indicated that all cases of mild acute pancreatitis (AP) displayed true diffusion restriction, as evidenced by high signal intensity in high b-value images and low signal in the corresponding ADC map. On the other hand, facilitated diffusion was observed in 80% of patients diagnosed with necrotizing pancreatitis, which showed low signal in high b values and high signal in the corresponding ADC map. Thus, it was concluded that the sensitivity of high b values for detecting mild forms of AP was 100%, while for severe necrotizing forms of AP, it was 80%. These results align with a study conducted by Arora *et al*., in which 48 out of 50 patients with early-stage AP exhibited diffusion restriction [19]. Comparable results were observed in additional research assessing the significance of DWI in the diagnosis of AP [14, 17]. The quantitative evaluation of DWI analysis in this study revealed that the average ADC value for a normal pancreas was 1.54±0.05 x10-3 mm^2^/sec, which closely corresponds with the findings reported by Ma and colleagues [20]. In contrast, the mean ADC value for the pancreatitis group was 1.27±0.3×10−3 mm^2^/sec, while the average value for severe necrotizing pancreatitis was 1.99±0.06 x10-3 mm^2^/sec. Similar values were observed by de Freitas Tertulino *et al*., who reported a mean ADC value of 1.18×10−3 mm^2^/s in the pancreatitis group and 1.70×10−3 mm^2^/s in cases of severe necrotizing pancreatitis [21]. Hocaoglu E and colleagues also reported significantly lower ADC values in the AP group (1.46±2.80 ×10−3 mm^2^/s) compared to the normal group (1.69±2.26 ×10−3 mm^2^/s) [11]. Likewise, the studies conducted by Arora *et al*. and Li *et al*. revealed significant differences between patients and controls, thus validating the diagnostic utility of DWI [19, 22]. Consequently, our findings have validated the outcomes of earlier research regarding the average ADC values of both the healthy and acutely inflamed pancreas. This can be attributed to the presence of edema within the extracellular space, which restricts the flow of water molecules, resulting in diminished signal intensity on the ADC map and a lower ADC value in cases of acute edematous pancreatitis. Conversely, when pancreatic tissue experiences necrosis, the rupture of cell membranes permits the diffusion of water molecules, leading to an increase in signal intensity on the ADC map and a rise in the ADC value.

Over the years, a variety of scoring systems have been introduced to assess the severity of acute pancreatitis. The revised Atlanta classification is regarded as one of the most well-structured systems for precisely defining acute pancreatitis, standardizing terminology among different specialties, and facilitating treatment planning [23]. Nevertheless, there is insufficient data available in the literature on the association between DW-MRI and the Revised Atlanta classification. Based on the Revised Atlanta Classification, our research indicated that 58.8% of the patients experienced moderate acute pancreatitis (AP), 29.4% had mild AP, and 11.8% suffered from severe AP. A significant correlation was found between ADC values and the revised Atlanta classification in distinguishing normal pancreas from AP (*p* <0.001). The mean pancreatic ADC value in the pancreatitis group (1.27±0.3×10−3 mm^2^/s) was notably lower than that in the normal group (1.54±0.05 x10-3mm^2^/sec) (*p* <0.001). This finding is consistent with the report by Thomas S and colleagues, who observed similar results [14]. Additionally, our research revealed a significant difference in the mean ADC value for severe necrotizing pancreatitis (1.99±0.06 x10-3 mm^2^/s) compared to non-severe cases (1.34±0.21 x10-3 mm^2^/s) (*p* < 0.001). Conversely, the mean ADC values for mild edematous (1.14±0.06 x10-3 mm^2^) and moderate edematous (1.18±0.16 x10-3 mm^2^) pancreatitis did not show a significant difference (*p* =0.349). The challenge in distinguishing between mild and moderate acute pancreatitis cases using DWI may stem from the nature of ADC values, which reflect overall water mobility within a tissue and are influenced by both molecular diffusion and microcirculatory blood perfusion; thus, they may not entirely capture the tissue characteristics [24]. This situation may open avenues for additional research into intravoxel incoherent motion diffusion, which could provide a more effective evaluation of inflammatory changes in the pancreas.

## 5 STUDY LIMITATIONS

The limitations of our study encompass a small sample size and insufficient diversity among the patients concerning the etiologies of acute pancreatitis (AP). Consequently, there was no correlation identified with a particular etiology of AP, such as autoimmune pancreatitis or hypertriglyceridemia, owing to the limited number of patients presenting these underlying conditions. Future investigations involving larger sample sizes, multicenter trials, and meta-analyses could establish a consistent ADC value threshold that would improve the diagnostic effectiveness of this method and address the discrepancies in numerical cutoffs observed across various institutions.

## CONCLUSION

Diffusion-weighted MRI provides both qualitative and quantitative evaluations, making it a concise modality for the early determination of and staging of acute pancreatitis. It is typically fast, radiation-free, offers greater convenience with reduced contrast exposure, and provides better detection of early cases compared to traditional CT examinations. This process aids in identifying the optimal management approach and accurately forecasting patient outcomes. Consequently, a transition from contrast-enhanced CT to MRI for evaluating the severity of acute pancreatitis (AP) is recommended.

## Figures and Tables

**Fig. (1):**
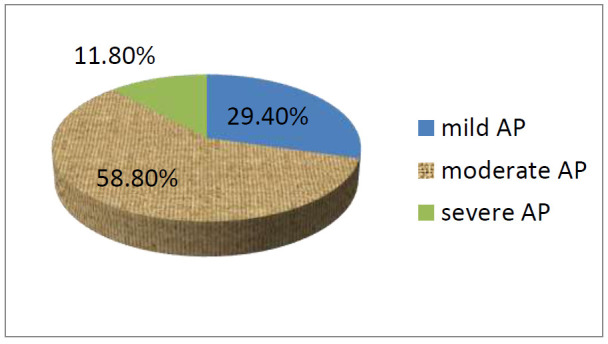
Severity of AP according to the revised Atlanta classification.

**Fig. (2):**
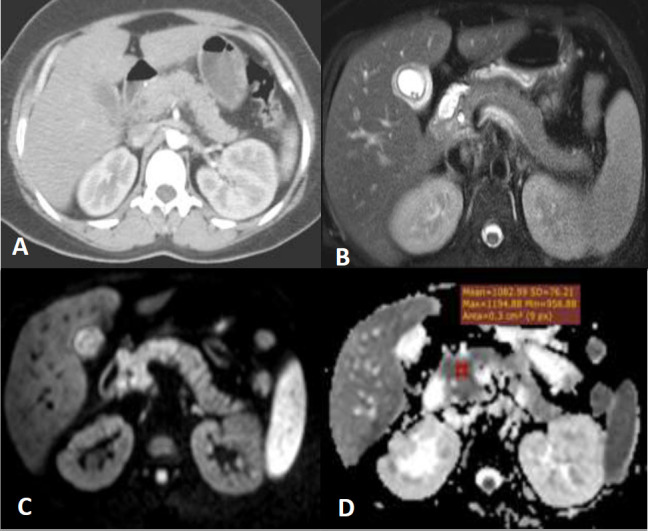
A 35-year-old female patient presented with epigastric abdominal pain radiating to the back and elevated serum amylase. (**A**) Enhanced CT showed equivocal features for acute pancreatitis. (**B**) T2WI demonstrated an isointense signal of the pancreatic tissue. (**C** & **D**) Show high signal on DWI B800 and low signal on the ADC map, indicating restricted diffusion in the pancreatic head and body. ADC value = 1.082 × 10^−3^ mm^2^/sec. The case was diagnosed as interstitial edematous pancreatitis.

**Fig. (3):**
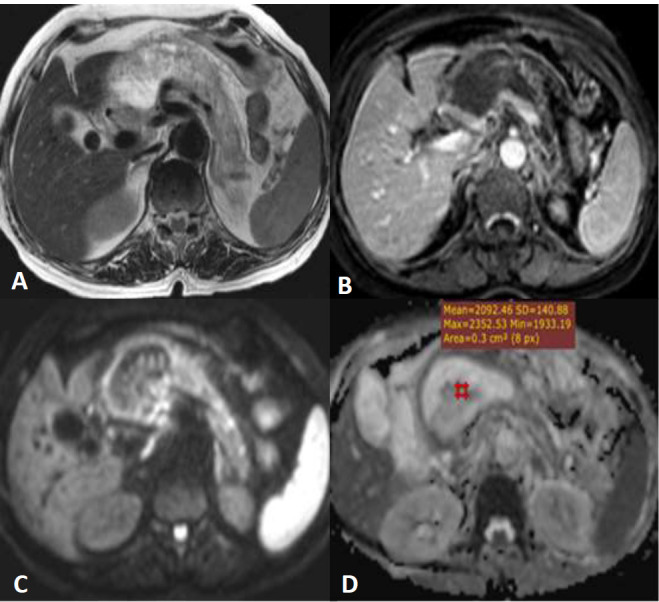
A 46-year-old female patient presented with intense epigastric pain and vomiting, accompanied by elevated serum amylase levels. (**A**) T2WI showed a hyperintense signal of the pancreatic body. (**B**) Enhanced T1WI fat sat shows no enhancement of the pancreatic body. (**C** & **D**) show facilitated diffusion of the pancreatic body at DWI B 800 and ADC map with ADC value =2.092 X 10-3mm^2^/sec. The case was diagnosed as necrotizing pancreatitis.

**Fig. (4):**
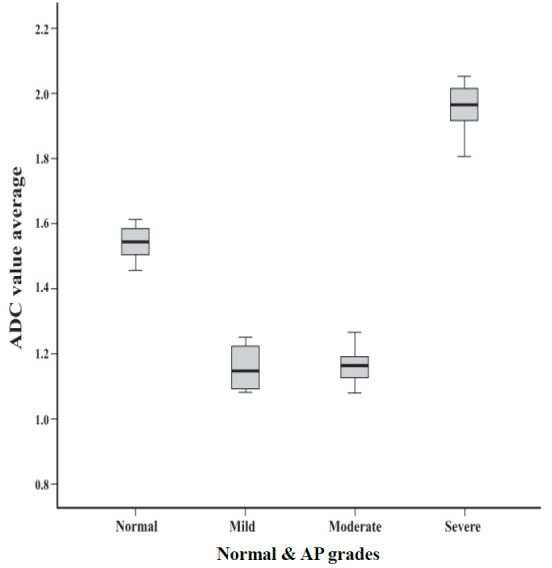
Box and whisker plot graph shows ADC value of normal pancreas, mild, moderate, and severe pancreatitis.

**Fig. (5):**
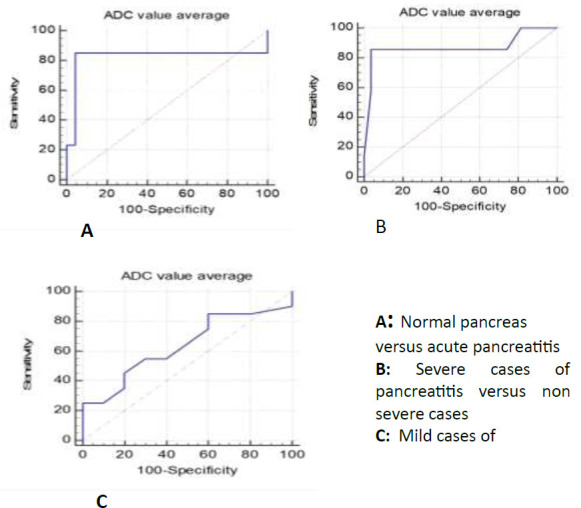
Receiver operating characteristic (ROC) curve analysis.

**Table 1:** Shows revised Atlanta classification.

**Severity**	**Complications**		
	**Organ Failure**	**Local Complications**	**Systemic Complications**
Mild	−	−	−
Moderately severe	Transient (<48 hrs)	+	+
Severe	Persistent (>48 hrs); single or multiple organ failure	+	+

**Table 2:** Parameters of different MRI sequences.

**Sequence** **Parameter**	**Coronal T2**	**Axial T2**	**Axial T2 SPAIR**	**Axial T1 FFE**	**Axial T1 Fat Sat.**	**DWI**
**Repetition time** **(TR)(ms)**	526	1670	768	260	4.1	1348
**Echo time(TE)(ms)**	80	100	80	4.6	1.96	65
**Slice thickness(mm)**	5	5	7	5	1.3	7
**Field of view (FOV)(mm)**	425	375	273	375	375	375
**Matrix**	304X264	400X224	304X209	240X118	180X142	124X100
**Flip angle (FA) (°)**	90	90	90	80	10	90
**Acquisition time(min)**	1:06	4:03	2:36	15 sec	11 sec	6:30

**Table 3:** Correlation between ADC values in different AP grades according to the revised Atlanta classification.

**According to the Revised Atlanta Classification**	***p*-value**
Normal *versus* mild	<0.001**
Normal *versus* moderate	<0.001**
Normal *versus* severe	<0.001**
Normal *versus* acute pancreatitis	<0.001**
Mild *versus* moderate	0.349
Mild *versus* severe	<0.001**
Moderate *versus* severe	<0.001**
Severe *versus* not severe	<0.001**

Note: ** Statistically significant difference (*p* <0.05).

**Table 4:** Shows AUC, Cutoff value, sensitivity, specificity, PPV, NPV and accuracy between the studied groups.

-	**AUC**	**Cutoff**	**Sensitivity**	**Specificity**	**PPV**	**NPV**	**Accuracy**
Normal *versus* acute pancreatitis	0.827	≤1.26	85.3	95.8	96.7	82.1	90.6
Severe pancreatitis *versus* not severe cases	0.870	>1.805	85.7	96.3	85.7	96.3	91.1
Mild *versus* moderate pancreatitis	0.635	>1.16	45.0	80.0	81.8	42.1	62.5

## Data Availability

All data generated or analyzed during this study are included in this published article.

## LIST OF ABBREVIATIONS

AP: Acute pancreatitis
DW: Diffusion-weighted
MRI: Magnetic resonance imaging

## STANDARDS OF REPORTING

STROBE guidelines were followed.
